# Profiling the resting venom gland of the scorpion *Tityus stigmurus* through a transcriptomic survey

**DOI:** 10.1186/1471-2164-13-362

**Published:** 2012-08-01

**Authors:** Diego D Almeida, Katia C Scortecci, Leonardo S Kobashi, Lucymara F Agnez-Lima, Silvia R B Medeiros, Arnóbio A Silva-Junior, Inácio de L M Junqueira-de-Azevedo, Matheus de F Fernandes-Pedrosa

**Affiliations:** 1 Laboratório de Tecnologia e Biotecnologia Farmacêutica, Universidade Federal do Rio Grande do Norte, Av. Gal. Cordeiro de Farias, s/n, CEP 59010-180 Natal, RN, Brazil; 2 Laboratório de Biologia Molecular e Genômica, Universidade Federal do Rio Grande do Norte, Natal, RN, Brazil; 3 Centro de Biotecnologia, Instituto Butantan, Av. Prof. Vital Brazil, 1500, CEP 05503-900, São Paulo, SP, Brazil

## Abstract

**Background:**

The scorpion *Tityus stigmurus* is widely distributed in Northeastern Brazil and known to cause severe human envenoming, inducing pain, hyposthesia, edema, erythema, paresthesia, headaches and vomiting. The present study uses a transcriptomic approach to characterize the gene expression profile from the non-stimulated venom gland of *Tityus stigmurus* scorpion.

**Results:**

A cDNA library was constructed and 540 clones were sequenced and grouped into 153 clusters, with one or more ESTs (expressed sequence tags). Forty-one percent of ESTs belong to recognized toxin-coding sequences, with transcripts encoding antimicrobial toxins (AMP-like) being the most abundant, followed by alfa KTx- like, beta KTx-like, beta NaTx-like and alfa NaTx-like. Our analysis indicated that 34% of the transcripts encode “other possible venom molecules”, which correspond to anionic peptides, hypothetical secreted peptides, metalloproteinases, cystein-rich peptides and lectins. Fifteen percent of ESTs are similar to cellular transcripts. Sequences without good matches corresponded to 11%.

**Conclusions:**

This investigation provides the first global view of gene expression of the venom gland from *Tityus stigmurus* under resting conditions. This approach enables characterization of a large number of venom gland component molecules, which belong either to known or non yet described types of venom peptides and proteins from the Buthidae family.

## Background

Scorpion morphology has changed little over the last four hundred million years. In the other hand, they naturally developed venom glands as a special weapon used in prey and defense. *Tityus stigmurus* belongs to the *Buthidae* family, widely distributed around the world and comprising all the species considered of medical interest [1]. In Brazil, scorpions from the genus *Tityus* are responsible for most reported envenomation accidents, primarily *Tityus serrulatus, Tityus stigmurus* and *Tityus bahiensis*[2]. *T. stigmurus* is the main causal agent of scorpionism in the Northeast; its envenomation is often characterized by local symptoms, such as: pain (94.4%), hyposthesia (30%), edema (17.8%), erythema (17.8%) and paresthesia (15.6%) [3]. Nishikawa [4] reported that *T. stigmurus* venom is the most toxic (DL_50_ = 0.773mg/kg) when compared to *T. serrulatus* and *T. bahiensis*. Nevertheless, *T. serrulatus* is the only species that has been significantly studied.

In addition to their clinical relevance, scorpion venoms are known to contain a very complex mixture of biologically active compounds [5]. Of these, neurotoxins are the most studied and play a key role in the pathogenesis of scorpionism. These toxins are small peptides that interact with several types of ion channels, modifying the electrical activity of excitable cells [6]. The most widely known ion channels recognized by these molecules are Na^+^ channels [7], K^+^ channels [8], ryanodine sensitive Ca^2+^ channels [9], T-type Ca^2+^ channels [10,11] and Cl^-^ channels [12]. Their properties make these peptides useful as molecular and pharmacological tools for studying ion channels. Another noteworthy class of molecules present in the venom gland are antimicrobial peptides, which may be involved in ancient innate immunity [13]. There are an estimated 150,000 distinct polypeptides found in the approximately 1500 known scorpion species worldwide [14], representing a broad scope for drug research and development.

The venoms of *T. stigmurusT. serrulatus* and *T. bahiensis* have similar toxic components and display a high degree of cross reactivity between specific antiserums [15-18]. Earlier studies reported the sequence of some *T. stigmurus* toxins*,* homologous to the previously known gama, III-8 and IV-5 toxins from *T. serrulatus*. These were named Tst-1, Tst-2 and Tst-3, respectively, [15] and are toxic to mice, recognizing Na-channels through different modes of action [19,20]. Holaday et al. [21] purified butantoxin, a K-channel blocker from the three medically important *Tityus* species mentioned above. Potassium channel toxins were also predicted in *Tityus stigmurus* venom using a proteomic approach [22].

Although the scorpion venom repertoire has been extensively investigated by PCR-based methods conducted with cDNA libraries [23-25], this strategy, in addition to cloning, isolation and characterization procedures, is limited by the specificity of the PCR primers used. In recent years, the number of proteomic and transcriptomic analyses performed has increased [26-35], since they are better able to assess venom diversity. Thus, in addition to known venom peptides and proteins, non yet described molecules can also be obtained. Moreover, transcriptomics has the advantage of providing insight into biological processes occurring in venom gland cells.

Previous investigations have used milked scorpion glands to achieve an enriched toxin library [26,27,29,30]; however, only one used a so called “replete” venom gland not actively engaged in regenerating venom [28]. Few scorpion nucleotide sequences are currently deposited in public databases, particularly for the *Tityus* genus, despite its clinical importance. The present study describes the transcriptomic expression of *T. stigmurus* scorpion from non-stimulated venom glands, using specimens collected in the urban area of Natal, Brazil.

## Results and discussion

### Overview of ESTs from the venom gland of *T. stigmurus*

**Figure 1:**
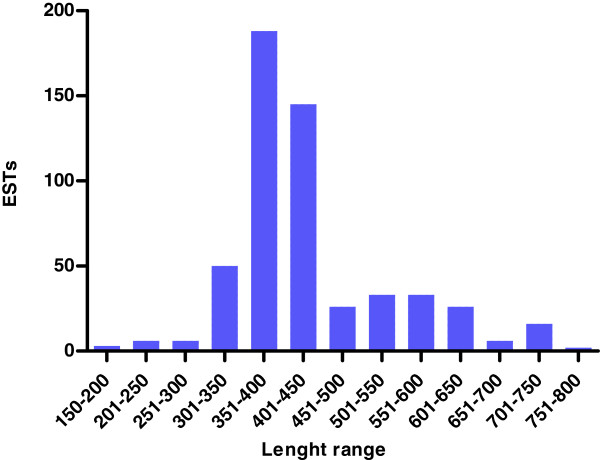
**Length distribution of*T. stigmurus*venom gland ESTs.** A total of 540 clones were analyzed. Abscissa is the length of sequences in 50 bp intervals, whereas the total number of ESTs for each interval is shown in the Y-coordinate. The average length of ESTs was 441 pb.

After poor-quality sequences were discarded, the remaining 540 high-quality ESTs were used to analyze gene expression profile in the venom gland of *T. stigmurus*. ESTs were grouped into 153 clusters, 37 corresponding to ‘contigs’ and 116 to ‘singlets’ (Additional file 1: ESTs from *Tityus stigmurus*). As such, these clusters were considered putative unigenes, although some may still represent different segments of the same gene. All sequence data reported in this investigation have been submitted to the public database [GenBank: JK483709 - JK483861]. The average length of ESTs was 441 pb and length distribution is shown in Figure1.

Sequence clusters were denominated TSTI0001C to TSTI0037C, for clusters with more than one EST, or TSTI0038S to TSTI0153S for those containing only one EST. When compared to data from GenBank and dbEST, we found that of the 153 clusters (540 clones) identified, 113 exhibited significant similarities to known cDNA and protein sequences. This corresponds to 486 clones (90%); the remaining 54 (10%) were not identified and defined as “no hit”. Six clusters exclusively matching mitochondrial DNAs, mRNAs and ribosomal RNAs were also found and excluded from quantitative analyses.

**Figure 2:**
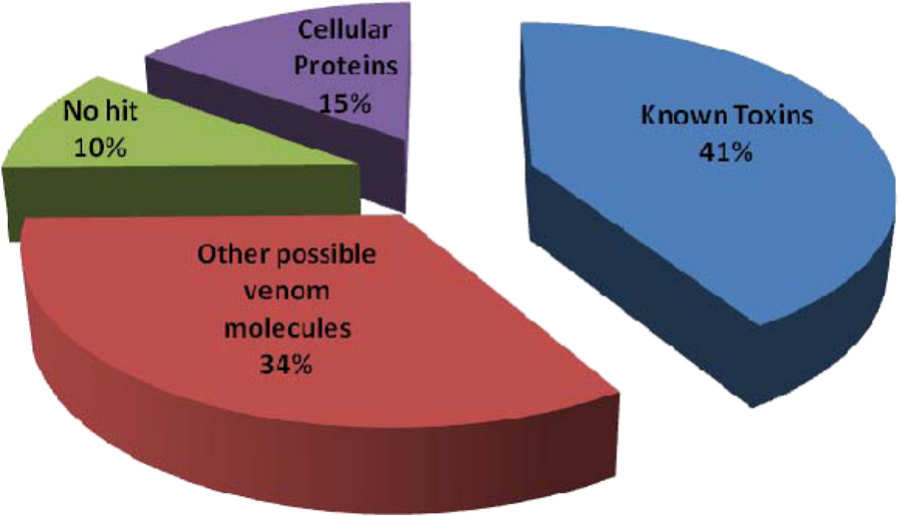
**Functional classification of transcripts from*Tityus stigmurus*venom glands.** Graph showing the relative proportion of different types of transcripts: ‘Known Toxins’, ‘Other possible venom molecules’, ‘Cellular Proteins’, and no-match sequences (‘No Hit’) [A].

Clusters were organized into three categories: proteins similar to well-known venom toxins, molecules with probable toxic activity and proteins associated with cellular functions. Figure2 shows that ‘known toxins’ represent 41% of all cDNAs (28 clusters with 222 clones) and 45.87% of defined sequences, while ‘other possible venom molecules’ correspond to 34% of all cDNAs (27 clusters with 180 clones) and 37.2% of defined sequences. ‘Cellular proteins’ represent 15.24% of the total number of clones and 17% of the matching clones. The remaining sequences are transcripts that do not match database sequences (10% of total clones, with 34 clusters and 54 clones).

Table1 shows the twelve most abundant transcript groups, all related to ‘known toxins’ or ‘other possible venom molecules’ products, except for the “Unknown Function” and “arginine kinase” groups.

**Table 1:** **Identification of high-abundance transcripts present in*T. stigmurus*venom glands**

**Groups**	**Number of clusters**	**Number of clones**	**Clones/clusters**	**% of total**	**Putative identification**
1	8	147	18.38	27.22	Anionic peptide*
2	9	136	15.11	25.19	AMP- like*
3	10	48	4.80	8.89	alfa KTX-like*
4	3	25	8.33	4.63	beta KTX-like*
5	8	20	2.50	3.70	hypothetical secreted peptide*
6	7	8	1.14	1.48	Unknown Function^1^
7	2	8	4.00	1.48	arginine kinase
8	6	7	1.25	1.30	Metalloprotease*
9	1	6	6.00	1.11	Hypotensin*
10	3	5	1.67	0.93	beta NaTX-like*
11	2	2	1.00	0.37	alfa NaTX-like*
12	1	2	2.00	0.37	cystein-rich peptide*

The (*) indicates detection of a putative signal peptide, predicted using the SignalP 3.0 program (http://www.cbs.dtu.dk/services/SignalP/). ^1^ Signal peptides were detected in some clusters.

### Known toxins

**Figure 3:**
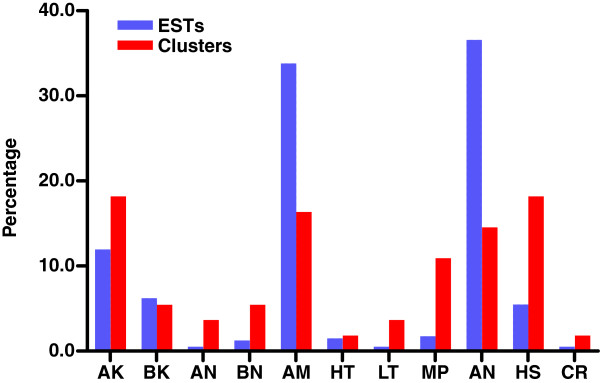
**Functional classification of the “Known toxins” and “Other possible venom molecules” categories by relative abundance, considering only these categories.** Acronyms are designated as follows: AK, alfa KTX; BK, beta KTX; AN, alfa NaTX; BN, beta NaTX; AM, Antimicrobial peptides; HT, Hypotensins; LT, Lectins; MP, Metalloproteases; AN, Anionic peptides; HS, Hipothetical secreted peptides; CR, Cystein-rich peptides. The blue and gray bars show the classification groups according to EST and cluster abundance, respectively.

Six known toxin-related groups were identified in the *Tityus stigmurus* venom gland transcriptome: α-KTX-like, β-KTX-like, α-NaTX-like, β-NaTX-like, Hypotensins and Antimicrobial Peptides. Figure3 exhibits the repertoire of known toxins found in this investigation.

#### Potassium channel toxins (α-KTX, β-KTX)

**Figure 4:**
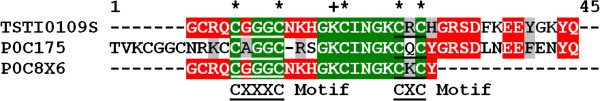
**Alignment of the amino acid sequence for TSTI0109S, from*T. stigmurus*venom glands, with known potassium channel toxins (α-KTX).** Residues are numbered according to the aligned potassium channel toxins (α-KTX) sequences and dots represent gaps introduced to improve alignment. The putative signal peptide is omitted. Conserved cystein residues are indicated by asterisks. The key lysine for blocking activity is detached by a cross. The CXXXC and CXC motifs conserved among scorpion neurotoxins are underlined. Green, red and gray indicate amino acids that are identical, conserved or similar, respectively. The abbreviation and GenBank accession number for the aligned potassium channel toxins sequences are: TsPep2, *Tityus serrulatus* potassium channel toxin (P0C175); Tst-17, *Tityus stigmurus* potassium channel toxin (P0C8X6).

**Figure 5:**
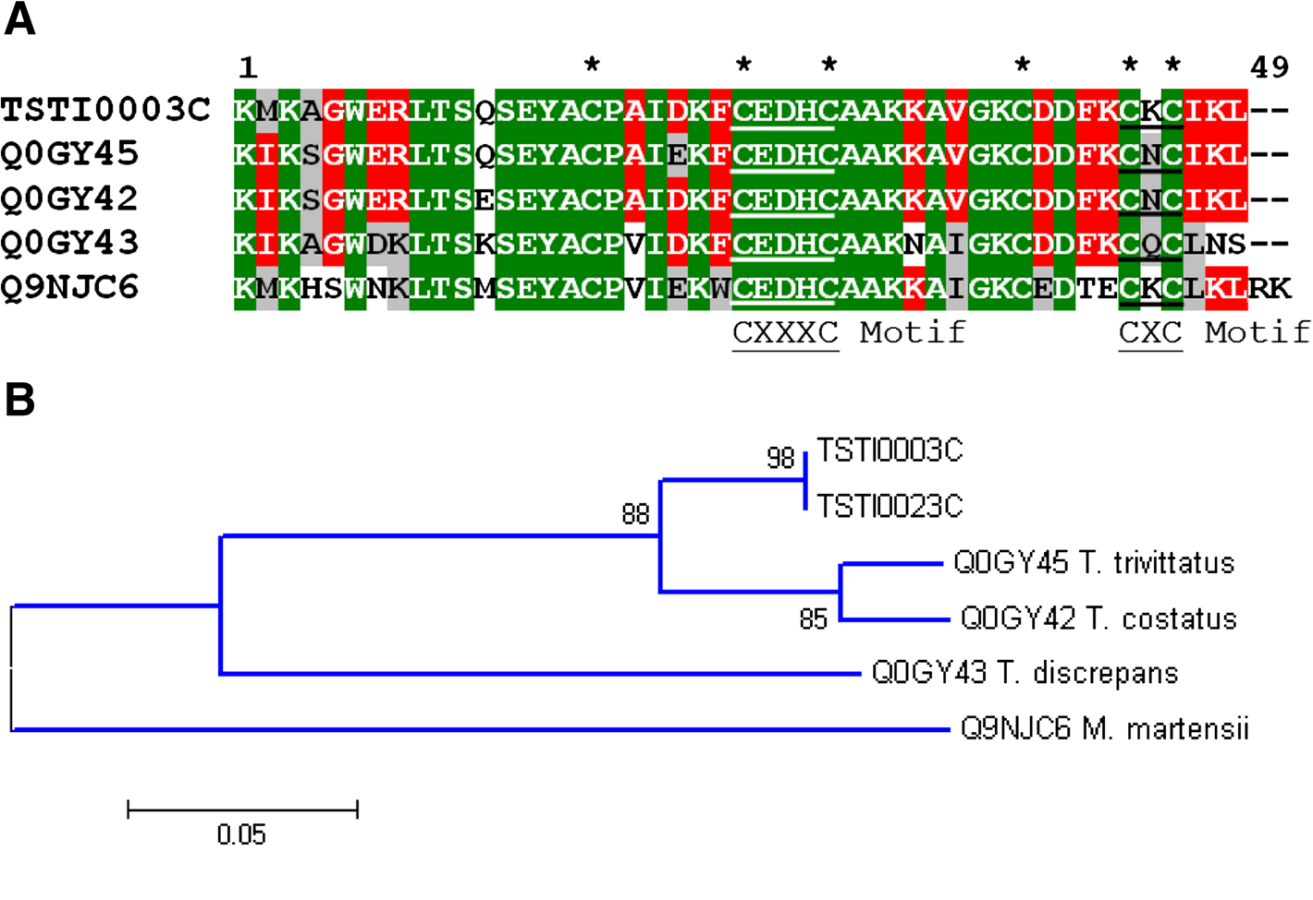
**Alignment of the amino acid sequence for TSTI0003C, from*T. stigmurus*venom glands, with known potassium channel toxins (β-KTX).** Residues are numbered according to the aligned potassium channel toxins (β-KTX) sequences and dots represent gaps introduced to improve alignment. The putative signal peptide is omitted. Conserved cystein residues are indicated by asterisks. The CXXXC and CXC motifs conserved among scorpion neurotoxins are underlined. Green, red and gray indicate amino acids that are identical, conserved or similar, respectively. The abbreviation and GenBank accession number for the aligned potassium channel toxins sequences are: TtrKIK, *Tityus trivitattus* potassium channel toxin (Q0GY45); TcoKIK, *Tityus costatus* potassium channel toxin (Q0GY42); TdiKIK, *Tityus discrepans* potassium channel toxin (Q0GY43) and BmTXKβ, *Mesobuthus marteensi* potassium channel toxin (Q9NJC6) [**A**]. Dendogram of β-KTX peptides sequences from scorpion venoms [**B**].

Potassium channel toxins have been reported in almost all scorpion species studied. They are 23 to 64 amino acid residues in length and densely packed by three or four disulfide bridges [8]. Ten clusters (48 clones) were identified as putative α-KTX toxin precursors, the second most abundant of the ‘known toxins’. Two (TSTI0075S and TSTI0109S) were similar to the short toxin structural class found in *Tityus serrulatus* venom, consisting of TsPep1, TsPep2 and TsPep3 (*T. serrulatus* peptide 1, 2 and 3) [36]. TSTI0075S and TsPep2 contain 68 amino acid residues and the predicted signal peptide differs in only two amino acids, meaning it may encode the same mature peptide. TSTI0109S has a shorter sequence, with a length of 62 amino acids, and its predicted mature sequence shares 91% identity with Tst-17 (Figure4) previously recorded in *T. stigmurus* venom using a proteomic approach [22]. Two other clusters (TSTI0122S, TSTI0140S) exhibited 68% and 93% identity with the *T. costatus* toxin α-KTX 4.5 [37]. Other cysteine-rich sequences showed homology to alpha-KTX peptides, with TSTI0016C the most representative containing 34 clones. TSTI0016C is 60% identical to “cysteine-rich peptide clone 2”, a putative toxin from *T. costatus*[37]. In regard to sequences matching β-KTX, we obtained 3 related clusters (25 clones), all of which could be aligned to the “orphan” components TcoKIK, TtrKIK, TdiKIK and BmTXKβ found in *T. costatusT. trivitattusT. discrepans* and *Mesobuthus marteensi*, respectively (Figure5A). These components were assumed to be authentic orthologous genes and denominated as “orphan” since their function is not well defined [38].

TSTI0003C may be a new member of this orthologous genes family, displaying greater similarity to β-KTX genes from *T. costatus* (gb|Q0GY45) and *T. trivitattus* (gb|Q0GY42) when compared to the other family members (Figure5B).

#### Sodium channel toxins (α-NaTX, β-NaTX)

**Figure 6:**
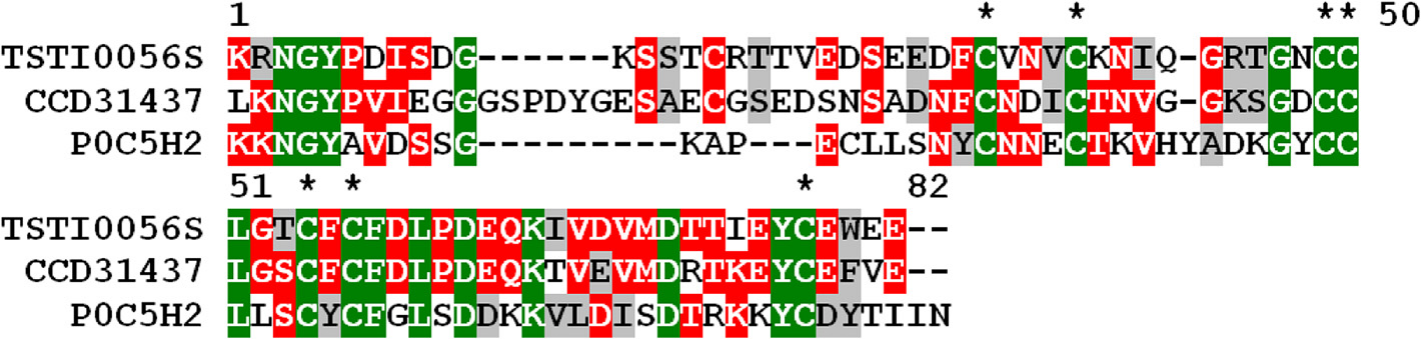
**Alignment of the amino acid partial sequence for TSTI0056S, from*T. stigmurus*venom glands, with known sodium channel toxins (β-NaTX).** Residues are numbered according to the aligned sodium channel toxins (β-NaTX) sequences and dots represent gaps introduced to improve alignment. The putative signal peptide is omitted. Conserved cystein residues are indicated by asterisks. Green, red and gray indicate amino acids that are identical, conserved or similar, respectively. The abbreviation and GenBank accession number for the aligned sodium channel toxins sequences are: Tpa8, *Tityus pachyurus,* sodium channel toxin (CCD31437); IsomTx2, *Isometrus vittatus****,*** potassium channel toxin (P0C5H2).

The venom of scorpions from the Buthidae family contains abundant sodium channel toxins, in contrast to non-Buthidae scorpions. These neurotoxins are involved in envenomation lethality [18,31]. However, some reports focusing on molecular analysis of the scorpion venom repertoire have proved that variations in venom composition may occur due to uncontrolled external factors, including depletion and environmental conditions [29,39]. In addition, considering the relative number of clusters, low representation of sodium channel toxins in Buthidae scorpions was previously found in the *Lychas mucronatus* transcriptome, in which sodium toxins accounted for 3.2% of clusters [29]. This finding is similar to that of our study on *Tityus stigmurus* transcriptome, in which we obtained only two clusters (2 clones) encoding for α-NaTX-like sequences and 3 (5 clones) for β-NaTX-like sequences (Figure6). This fact may be associated with lower lethality of *Tityus stigmurus* human envenoming when compared to *Tityus serrulatus* accidents.

Another similar case was that of *Hottentota judaicus* scorpion “resting” venom glands, where sodium channel toxins were underrepresented and the αNaTx:βNaTx ratio inversed [28]. Sequences matching Tst-1 (TSTI0051S), Tst-2 (TSTI0033C) and Tst-3 (TSTI0151S) were also found in this group.

#### Hypotensins

**Figure 7:**
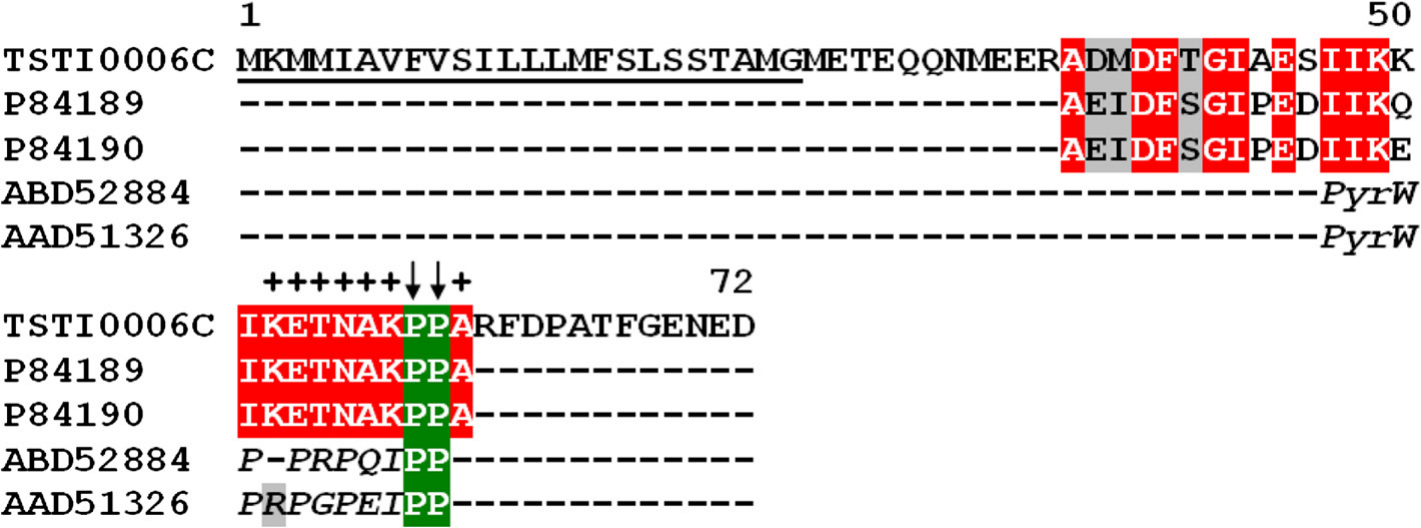
**Alignment of the amino acid sequence for TSTI0006C, from*T. stigmurus*venom glands, with known hypotensins.** Residues are numbered according to the aligned hypotensin sequences and dots represent gaps introduced to improve alignment. The underlined amino acid sequence indicates the putative signal peptide. Crosses indicate the C-terminal region probably involved in the hypotension effect. The similar BPP amino acid signature represented by a proline doublet is indicated by arrows. *Pyr* is the N-terminal pyroglutamic acid residue typical of snake BPPs. Green, red and gray indicate amino acids that are identical, conserved or similar, respectively. The abbreviation and GenBank accession number for the aligned hypotensin sequences are: Hypotensin-I, *Tityus serrulatus* (P84189), Hypotensin-II, *Tityus serrulatus* (P84190), BPP, *Lachesis muta* (ABD52884) and BPP, *Bothrops jararaca* (AAD51326).

Bradikynin Potentiating Peptides (BPPs), peptides with hypotension properties, have been described in different animal venoms, including snakes, frogs and scorpions [40-43]. These peptides usually inhibit angiotensin converting enzymes (ACEs) and the breakdown of endogenous vasodilator bradykinin, leading to reduced systemic blood pressure [44]. Recently, Verano-Braga [45] discovered a group of BPPs in *Tityus serrulatus* venom (*T. serrulatus* Hypotensins: TsHpt-I, TsHpt-II, TsHpt-III and TsHpt IV) containing 24–25 amino acid residues. They display bradykinin-potentiating activity, without ACE inhibition, and their anti-hypertension activity appears to be caused by nitric oxide (NO)-dependent mechanisms. Interestingly, we identified one cluster (6 clones) showing high identity with the abovementioned hypotensins in the middle-region. The TSTI0006C cluster encodes a precursor with 72 amino acid residues and a putative 24 amino acid-long signal peptide (Figure7). It is important to note that pharmacological activity in these peptides seems to be located towards the C-terminal, suggesting a post-translational modification in this region since the predicted mature peptide has 48 amino acid residues [45].

#### AMPs (antimicrobial peptides)

**Figure 8:**
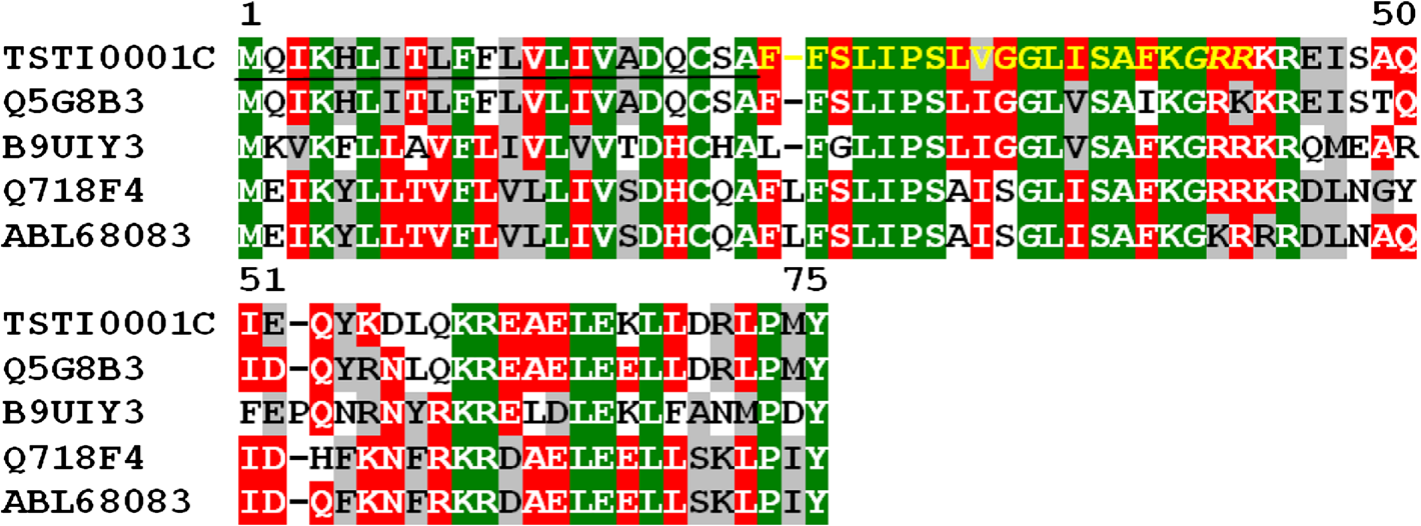
**Alignment of the amino acid sequence for TSTI0001C, from*T. stigmurus*venom glands, with known sequences of antimicrobial peptides.** Residues are numbered according to the aligned antimicrobial peptide sequences and dots represent gaps introduced to improve alignment. The underlined amino acids indicate the putative signal peptide. A possibly promature peptide is shown in yellow font and the putative post-translational signal GRR is in italics. Green, red and gray indicate amino acids that are identical, conserved or similar, respectively. The abbreviation and *GenBank* accession number for aligned antimicrobial peptide sequences are: *Tityus costatus* antimicrobial peptide (Q5G8B3), Mucroporin, *Lychas mucronatus* (B9UIY3), Bmkb1, *Mesobuthus martensii* (Q718F4) and Caerin-2 *Mesobuthus eupeus* (ABL68083).

Antimicrobial peptides are commonly found in scorpion transcriptomes [26-28,30]. They play an important role in innate immune systems and may depolarize neuronal cells inducing prey immobilization, as well as potentiate the action of other neurotoxins [46]. Surprisingly, AMPs were the most abundant category among toxins and the second when considering the entire transcriptome (136 clones, 9 clusters). High expression levels in AMPs were previously reported in *Lychas mucronatus* scorpions on Hainan, a hot, humid island in Southern China. These characteristics could cause greater susceptibility to pathogenic microbial infections [29]. Interestingly, a similar climate is found in the city where *T. stigmurus* specimens were collected. In addition, *T. stigmurus* are frequently found in sewage pipes hunting for prey, mainly cockroaches. It is therefore not coincidence that many envenomation cases occur in bathrooms. Thus, effective defenses are needed in this environment. TSTI0001C is the most representative transcript in this category (Figure8).

### Other possible venom molecules

Some transcripts found in *T. stigmurus* venom glands resembled putative molecules with potential toxic activity and were therefore classified as ‘other possible venom molecules’. Five groups fit into this category: Lectins, Metalloproteases, Anionic Peptides, Hypothetical Secreted Peptides and Cystein-Rich peptides.

#### Lectins

Lectins have not been reported for scorpions in transcriptomic, proteomic or related approaches. As such, to the best of our knowledge, there are no scorpion lectin sequences currently deposited in public databases, although lectins have been isolated from scorpion venom and hemolymph using chromatographic procedures [47,48]. Despite the lack of information on lectins in scorpion venoms, they have been studied in other venomous animals, such as fish, snakes and spiders [49-51] and may be involved in innate immunity. Our library contains two clusters (TSTI0068S and TSTI0118S) with arthropod lectin-like sequences.

#### Anionic peptides

**Figure 9:**
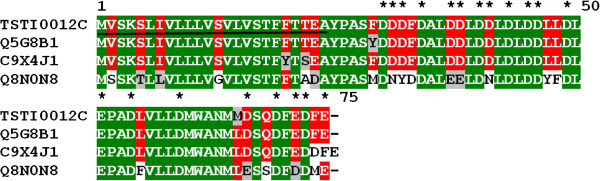
**Alignment of the amino acid sequence for TSTI0012C, from*T. stigmurus*venom glands, with known sequences of anionic peptides.** Residues are numbered according to aligned anionic peptides sequences and dots represent gaps introduced to improve alignment. The underlined amino acids indicate the putative signal peptide. Conserved acidic residues (D and E) are indicated by asterisks. Green, red and gray indicate amino acids that are identical, conserved or similar, respectively. The abbreviation and GenBank accession number for aligned anionic peptide sequences are: *Tityus costatus* anionic peptide (Q5G8B1), *Tityus discrepans* anionic peptide (C9X4J1), *Tityus martensii* anionic peptide (Q8N0N8).

Anionic peptide precursors are molecules with high acidic amino acid content. Unexpectedly, this category exhibits the most expressed transcripts (147 clones and 8 clusters). Anionic peptides have been recorded in both Buthidae and non-Buthidae scorpions [24,29-31,37,52], although they seem much more abundant in the former. Their function remains unclear, but one hypothesis suggests two possible roles: to help balance the pH value of the venom solution, since most scorpion venom peptides are basic, or to act synergistically with other peptides [24] (Figure9).

#### Metalloproteases

Alhough scorpion venom research has focused primarily on neurotoxic peptides, proteolytic activity has also been described [53,54]. Two types of proteases have already been characterized in scorpion venom glands: serineproteases (SPSVs) and metalloproteases [27,28,55]. Serine- and metalloproteases were also detected in venom transcriptomic analysis of other animals [51,56]. Despite the lack of transcripts similar to SPSVs, metalloproteases are significantly represented by 6 clusters (7 clones). Four of these are similar to antareases, a venom protein from *T. serrulatus*. Antarease is a divalent ion-dependent protease that cleaves vesicle-associated membrane proteins (VAMPs) at specific sites, leading to significant alterations in vesicular transport and secretory mechanisms. This action may be involved in pathogenesis mechanisms, including acute pancreatitis induction [57]. An additional two clusters encode for putative M13 metalloprotease and angiotensin converting enzymes from the *Hottentotta Judaicus* scorpion.

#### Hypothetical secreted peptides

**Figure 10:**
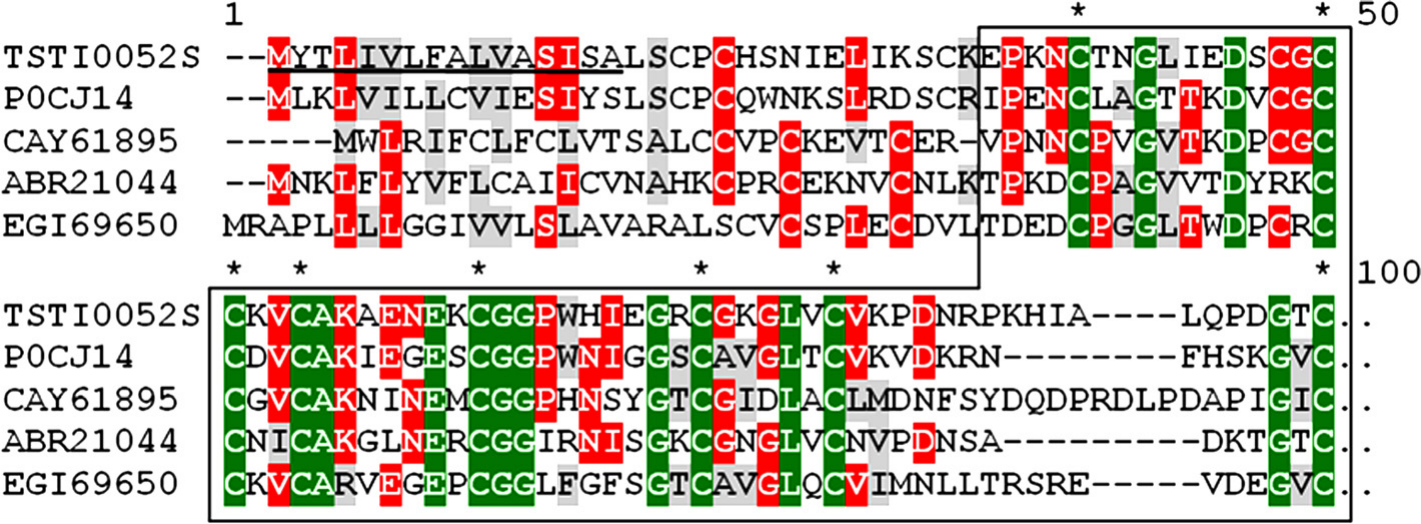
**Alignment of the amino acid sequence for TSTI0052S, from*T. stigmurus*venom glands, with known sequences of IGFBP (insulin-like growth factor-binding protein).** Residues are numbered according to aligned IGFBP sequences and dots represent gaps introduced to improve alignment. The underlined amino acids indicate the putative signal peptide. Conserved cystein residues are indicated by asterisks. The IGFBP domain is highlighted by a box. Green, red and gray indicate amino acids that are identical, conserved or similar, respectively. The GenBank accession number for aligned IGFBP sequences are: *Lychas mucronatus* (P0CJ14), *Mesobuthus eupeus* (CAY61895), *Tityus discrepans* (ABR21044), *Acromyrmex echinatior* (EGI69650).

Several transcripts (10 clusters, 22 clones) were similar to hypothetical secreted peptides from other scorpions. The function of these peptides is unknown; however, according to our data, some clones appear to be conservative in scorpion and arachnid venom or salivary glands. Further characterization is needed for clones belonging to this category. An interesting finding is the significantly expressed contig TSTI022C (7 clones), showing 91% identity with a partial mass spectrometry protein sequence (peptide 9797) from a *Tityus stigmurus* venom proteomic analysis [22]. Another finding is IGFBP (insulin-like growth factor-binding protein) domain containing sequences [TSTI052S, TSTI0064S and TSTI0125S]. IGFBPs modulate the physiological actions of insulin-like growth factors (IGFs) in several types of tissues [58], including tumor cells [59-61]. Other scorpion and insect venom molecules also had IGFBP domains [29,31] (Figure10).

#### Cysteine-rich secretory peptides

Cystein-rich secretory peptides (CRISPs) are widely distributed in the animal, plant and fungal kingdoms, with variable primary sequences [62-64], including different animal venoms [28,62,65]. The SCP_CRISP-like domain containing sequence is a cluster with 2 clones (TSTI0017C), similar to other arachnid and insect cysteine-rich peptides. Interestingly, as with helothermine [66], lizard venom CRISPs block Ca^++^ transporting ryanodine receptors, while the opposite action is reported for neurotoxins belonging to the calcin family found in scorpions [67].

The first analysis of a non-Buthidae scorpion resulted in 147 high-quality ESTs, which allowed the authors to examine the molecular repertoire of the venom gland [26]. Similar approaches have been applied with Buthidae and non-Buthidae scorpions species, showing marked differences in diversity and repertoire of toxin-like sequences [68]. The venom components commonly found in transcriptomics are sodium channel toxins, potassium channel toxins, calcines, AMPs, BPPs, phospholipases A_2,_ anionic peptides and glycine-rich peptides. Of these, only calcines, phospholipases A_2_ and glycine-rich peptides were not found in this study. The main novelty of this investigation is to present some poorly or as yet undescribed transcripts in scorpion venoms such as: lectins, metaloproteases, cystein-rich peptides and hypothetical secreted proteins.

Scorpion venom proteome studies have been previously carried out, although most components have not been sequenced [27,32,33,35]. Thus, a comparative proteomic analysis with other scorpion venoms is difficult to obtain. Nevertheless, the following transcripts match proteins found in *Tityus sp*. venom itself, using either proteomics or isolation and characterization approaches, as follows: TSTI0022C (hypothetical secreted peptide, similar to Peptide 9797, gb|P0C8X1), TSTI0051S (sodium channel toxin, similar to Tst1, gb|P56612), TSTI0033C (sodium channel toxin, similar to Tst2, gb|P68411), TSTI0151S and TSTI0048S (sodium channel toxin, similar to Tst3, gb|P0C8X5), TSTI0109S (potassium channel toxin, similar to Tst-17, gb|P0C8L2), TSTI0140S (potassium channel toxin, similar to Tst26 gb|P0CB56), TSTI0016C (potassium channel toxin, similar to Ts15 gb|P86270), TSTI0075S (potassium channel toxin, similar to TsPep2, gb|P0C175), TSTI0006C (Hypotensin-II, gb|P84190) and some Antareases-like sequences (gb|P86392).

### Cellular proteins

**Figure 11:**
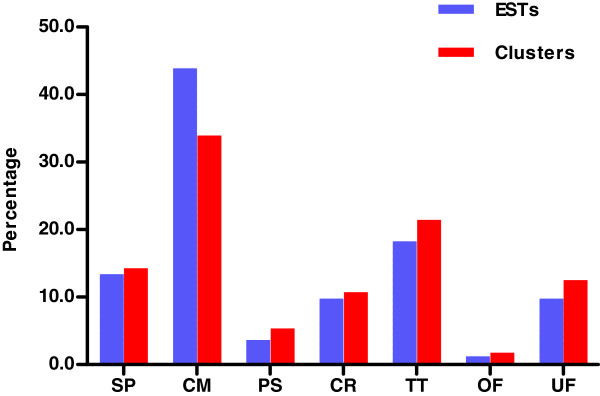
**Relative abundance of ESTs coding for cellular proteins.** Acronyms are designated as follows: SP, Structural proteins; CM, Cell Metabolism; PS, Processing and Sorting; CR, Cell Regulation; TT, Transcription and Translation; OF, Other Functions; UF, Unknown Functions. The blue and gray bars show the classification groups relative to EST and Cluster abundance, respectively.

In addition to transcripts predicted to be involved in venom toxicity, there are other housekeeping genes in *T. stigmurus* venom glands. Figure11 shows the 82 clones (15.24% of total) found in this study corresponding to ‘cellular proteins’, most of which are responsible for cellular metabolism (36 clones) and transcription/translation (15 clones).

The majority of transcripts involved in ‘cellular metabolism’ are similar to cytochrome c oxidase (3 clusters/ 12 ESTs), followed by arginine kinase (1 cluster/ 7 ESTs). Clusters TSTI0014C, TSTI0037C and TSTI0065S are similar to cytochrome c oxidase subunits 1 or 2. TSTI0014C resembles cytochrome c oxidase subunit 1 from *Centruroides noxius*. The terminal oxidase in respiratory chains of eukaryotes and most bacteria, is a multi-chain transmembrane protein located in the inner membrane of mitochondria and the cell membrane of prokaryotes (gb| AY995829.1). In parallel, cluster TSTI0013C (group of 7 ESTs) is similar to arginine kinase (represented in group 8, from Table1) from *Litopenaeus vanname* shrimp. Members of this enzyme family play a key role in animals as ATP-buffering systems for cells with high and variable rates of ATP turnover (gb| DQ975203.1).

The venom gland is an organ specialized in venom production, with almost 75% of the transcripts ‘known’ or ‘possible’ toxins. We can therefore assume that a substantial metabolic expenditure is required for this task, resulting in a natural demand for energy and transcription/translation functions. The next most abundant transcripts were ‘structural proteins’ (11 clones, 8 clusters) such as actin and myosin. The presence of these transcripts is not surprising, since telson is known to contain compressor muscles, whose function is to press the glands against the cuticle along its exterior lateral and ventral surfaces [1]. Cluster TSTI0018C (group of 3 ESTs) exhibits similarity with myosin light chain 2 from the *Avicularia avicularia* spider, a Ca^2+^-binding protein (EF-Hand superfamily) (gb| 3DTP_E).

Other relevant categories were ‘cell regulation’ (6 clusters/ 8 ESTs), ‘processing and sorting’ (3 ESTs) and transcripts with unknown functions (7 clusters/ 8 ESTs). In the last, we found 5 clusters matching ‘hypothetical proteins’ from arachnids (*Ixodes scapularis* and *Tityus discrepans*).

Taken together, these results represent important clues for the characterization of cellular and molecular functions in scorpion venom glands. Moreover, the repertoire generated in this approach is relevant in highlighting the transcripts of *T. stigmurus* venom glands, contributing to the international “Genbank” database and allowing subsequent isolation and application of these molecules.

## Conclusions

The present study describes the profile of gene expression present in the venom glands of *Tityus stigmurus* scorpions using a transcriptomic approach. This profile shows a wide range of structural and functional putative molecules in *Tityus stigmurus* venom glands. Six known protein types were identified, including ‘potassium channel’ (sub-families α and β) , ‘sodium channel’ (subfamilies α and β), ‘hypotensins’ and ‘antimicrobial peptides’, and five atypical types of venom peptides and proteins, such as ‘lectins’, ‘anionic peptides’, ‘metalloproteases’, ‘hypothetical secreted peptides’ and ‘cystein-rich peptides’. This strategy confirms the highly specialized nature of scorpion venom glands as toxin producers, enabling the description, for the first time, of putative proteins involved in cellular processes relevant to venom gland function of *T. stigmurus*. In particular, transcripts encoding antimicrobial peptides and anionic peptides were the most representative transcripts in this database. The transcriptome of *T. stigmurus* did not show high expression of sodium channel toxins as one might expect from a Buthidae scorpion, primarily for subfamily α. This type of toxin is the most studied among scorpions from the genus *Tityus* and has often been related to the severity of poisoning. Its absence may be associated to environmental conditions, where more antibacterial defenses may be required than neurotoxins for prey, since food is abundant. It may also be a characteristic of scorpion venom-filled glands in a resting stage.

This database of scorpion molecules described here may be an important resource for the investigation and characterization of proteins or peptides potentially applicable in pharmaceutical research and biotechnology.

## Methods

### cDNA library construction

A cDNA library was constructed from total RNA extracted from four telsons. Specimens were collected in the urban region of Natal, Brazil. The 'total RNA isolation system' of Promega (Madison, WI) was used for RNA isolation. With this material, a full-length cDNA library was prepared using the *In-Fusion™ SMARTer™ cDNA Library Construction Kit* (CLONTECH Lab., Palo Alto, CA). The titre of the non-amplified cDNA library obtained was 2 × 10^4^ cfu/mL with 90% recombinant clones. Reverse transcription used *SMARTer II A* and *3' SMART CDS Primer II A* oligonucleotides*.* Next, *5' PCR Primer II A* was employed for PCR amplification. Resulting cDNAs were bidirectionally cloned in the *pSMART2IF* plasmid (all components were from the CLONTECH Lab, Palo Alto, CA). *Escherichia coli* DH10β cells were transformed with cDNA library plasmids and plated on Luria-Bertani agarose plates containing 100 μg/mL ampicilin.

### DNA sequencing and bioinformatic analyses

Random clones were grown in antibiotic selective medium for 22 h and plasmid DNA was isolated using alkaline lysis [69]. DNA was sequenced on an ABI 3100 sequencer, using a BigDye2 kit (Applied Biosystems, Foster City, CA) and the standard M13 reverse primer. In order to extract the high quality sequence region, ESTs were subjected to the Phred program, with a cutoff Phred score of 20 in a window length of 75 bases [70]. Sequences were processed by removing vector, adaptors and *E. coli* DNA sequences using CrossMatch [71]. High-quality ESTs were assembled into contigs, using the CAP3 program [72] set to combine only those sequences with at least 98% base identity. To assign annotation to the assembled ESTs (clusters), these sequences were searched against nr and nt (E values < 1e-05) for homologous comparison using BLASTX and BLASTN [73], supported by the Blast2GO [74]. Metadata and bibliographic information, when available, were manually inspected to assign putative functional classification of the cluster. Additionally, proteins coded by the clusters were grouped according to possible participation in the venom. Three categories were established: 'known toxins', 'other possible venom molecules' or 'cellular proteins' for proteins with best hits to well-known scorpion venom toxins, proteins with hits to non-scorpion toxin sequences exhibiting activities compatible with toxic venom action, and other products related to cellular functions without evidence of being toxins, respectively. The presence of conserved domains, using the nr protein database or SMART [75] and Pfam [76], was also used to guide functional attribution. The occurrence of signal peptide was predicted with the SignalP 3.0 program [77], using both neural networks (NN) and hidden Markov models (HMM). A secretory protein was considered when both methods showed a signal peptide according to their default parameters (mean S > 0.048 and mean D score 0.43 > in NN and signal peptide probability > 0.5 in HMM).

### Alignment and dendogram

Alignment was conducted with the Vector NTI Suite program (Informax). The dendogram of Figure4B was created with the neighbor joining method implemented in MEGA5.03 [78].

## Competing interests

The authors declare they have no competing interests.

## Authors’ contributions

DDA performed the cDNA library, conducted bioinformatic analysis and drafted the manuscript. KCS participated in cDNA library and drafted the manuscript. LSK carried out DNA sequencing. LFA-L drafted portions of the manuscript. SRBM drafted portions of the manuscript. AAS-J drafted portions of the manuscript. IdLMJ–d–A performed data processing, bioinformatic analysis and reviewed the manuscript. MdFF–P participated in its design and coordination, in data analyses and drafted the manuscript. All authors read and approved the final manuscript.

## Supplementary Material

Additional file 1 **ESTs from*Tityus stigmurus.*** Table containing additional information about all the clusters from the scorpion *Tityus stigmurus.* Click here for file
